# Genome Sequences of Three Nocardia cyriacigeorgica Strains and One Nocardia asteroides Strain

**DOI:** 10.1128/MRA.00600-19

**Published:** 2019-08-15

**Authors:** Florian Vautrin, Emmanuelle Bergeron, Audrey Dubost, Danis Abrouk, Christophe Martin, Benoit Cournoyer, Vanessa Louzier, Thierry Winiarski, Veronica Rodriguez-Nava, Petar Pujic

**Affiliations:** a UMR CNRS 5557, INRA 1418 Ecologie Microbienne, Université Lyon 1, Villeurbanne, France; b Observatoire Français des Nocardioses, Institut des Agents Infectieux (IAI), Centre de Biologie et Pathologie Nord, Hôpital de la Croix-Rousse, Lyon, France; c Agressions Pulmonaires et Circulatoires dans le Sepsis (APCSe), VetAgro Sup–Campus Vétérinaire de Lyon, Marcy l’Etoile, France; d UMR CNRS 5023, Laboratoire d’Ecologie des Hydrosystèmes Naturels et Anthropisés, Vaulx-en-Velin, France; Indiana University, Bloomington

## Abstract

We report four draft genome sequences of Nocardia spp. The strains are the Nocardia cyriacigeorgica DSM 44484 pathogenic type strain; two environmental isolates, Nocardia cyriacigeorgica EML446 and EML1456; and the Nocardia asteroides ATCC 19247 nonpathogenic type strain, with estimated genome sizes of 6.3 to 6.8 Mb. The study of these isolates will provide insight into physiology, evolution, and pathogenicity of Nocardia spp.

## ANNOUNCEMENT

Since Edmond Nocard first isolated Nocardia spp. in 1888 (1), 92 different species have been described (2). Most of them are pathogenic for human or animal infections worldwide. Nocardia spp. are Gram-positive, acid-fast actinobacteria and are ubiquitous in nature (2). Pathogenic *Nocardia* cells can infect different organs in humans, as well as disseminate from a primary infection site, such as the lungs, to distant organs and other sites, including the central nervous system (3). Nocardia cyriacigeorgica is one of the most often implicated species in human nocardiosis, including strain GUH-2 (4). This species, which derives from the Nocardia asteroides complex drug pattern type VI (5) and was described as *N. cyriacigeorgica* in 2001 (6), seems to be composed of three genotypes (2, 7–9).

Currently, only a few genomes of *N. cyriacigeorgica* strains have been entirely sequenced, and the genome of GUH-2, the model organism to study infections in animals, is the only one that is annotated (10). A genomic comparison of these three genotypes could untangle this clustering and maybe describe a new species within the former *N. asteroides* complex. Four strains have been sequenced, as follows: Nocardia asteroides type strain ATCC 19247, the type strain *N. cyriacigeorgica* DSM 44484, and two *N. cyriacigeorgica* environmental strains, EML446 and EML1456, isolated from infiltration basin urban sediments in France (coordinates 45°73′55.01″N, 4°95′74.65″E). Sediment suspensions were prepared according to Maldonado et al. (11), and serial dilutions were inoculated on brain heart infusion (BHI) agar containing cycloheximide.

Strains were grown in BHI medium according to Beaman and Maslan (12), and genomic DNA was extracted from 50 ml cell culture during the exponential-growth phase. Cells were collected by centrifugation and pellet solubilized in 8 ml of 10 mM Tris-HCl, 1 mM EDTA (pH 8.2), 5 mg/ml lysozyme, and 10 μg/ml RNase A. After a 1-h period of cell lysis, 8 ml of 2% SDS and 2 mg/ml of proteinase K were added and incubated at 55°C for 5 h. Protein and cellular debris were eliminated by adding 16 ml phenol-chloroform-isoamyl alcohol, 25:24:1 (vol/vol/vol), at pH 8.2. After centrifugation, genomic DNA was precipitated by adding 1.5 ml of 3 M sodium acetate (pH 5.2) and absolute ethanol. Genomic DNA was collected by centrifugation, and the DNA pellet was washed in 70% ethanol, air dried, and solubilized in 200 μl of Tris-EDTA (TE) buffer (pH 8).

The genomes were sequenced using Illumina MiSeq technology by GATC (Mulhouse, France) and Biofidal (Vaulx-en-Velin, France). Libraries with 2 × 300-bp and 2 × 125-bp reads were constructed for Biofidal and GATC, respectively. The reads were processed using Unicycler v0.4.3 (13), quality controls were assessed with FastQC and Trimmomatic, and contigs shorter than 200 bp were removed, resulting in genomes of 6.6 Mb for *N. asteroides* ATCC 19247^T^, 6.3 Mb for *N. cyriacigeorgica* DSM 44484^T^, 6.5 Mb for *N. cyriacigeorgica* EML446, and 6.8 Mb for *N. cyriacigeorgica* EML1456. The genomes were annotated on MicroScope (14). The characteristics of the draft genomes are summarized in Table 1. These genome sequences provide valuable data to study the ecology, evolution, pathogenicity, phylogeny, and physiology of Nocardia cyriacigeorgica complex species.

**TABLE 1:** Characteristics of draft genomes and accession numbers of *N. asteroides* ATCC 19247^T^ and *N. cyriacigeorgica* DSM 44484^T^, EML446, and EML1456

Isolate	BioProject no.	Accession no.	Genome size (Mbp)	G+C content (%)	No. of contigs	No. of reads	*N*_50_ value (Mbp)	Coverage level (×)	No. of CDS^*a*^	% coding proteins	No. of tRNAs	No. of rRNAs
*N. asteroides* ATCC 19247^T^	PRJNA542835	VBUS00000000	6.6	70.0	18	13,451,664	1.51	301	6,498	91.4	50	2
*N. cyriacigeorgica* EML1456	PRJNA542859	VBUU00000000	6.8	68.0	108	16,883,270	0.15	370	6,906	89.7	49	2
*N. cyriacigeorgica* EML446	PRJNA542857	VBUT00000000	6.5	68.2	41	18,841,320	0.61	433	6,531	89.91	51	3
*N. cyriacigeorgica* DSM 44484^T^	PRJNA542831	VBUR00000000	6.3	58.2	64	10,182,338	0.19	487	6,072	89.8	48	3

a CDS, coding sequences.

### Data availability.

This whole-genome shotgun project has been deposited at DDBJ/ENA/GenBank under the accession numbers listed in Table 1. The versions described in this paper are the first versions. Data for EML446 and EML1456 are available at the CRB-EML (http://eml-brc.org/ and https://brclims.pasteur.fr/brcWeb/).
